# MeCIPK23 interacts with Whirly transcription factors to activate abscisic acid biosynthesis and regulate drought resistance in cassava

**DOI:** 10.1111/pbi.13321

**Published:** 2020-01-19

**Authors:** Yu Yan, Wen Liu, Yunxie Wei, Haitao Shi

**Affiliations:** ^1^ Hainan Key Laboratory for Sustainable Utilization of Tropical Bioresources College of Tropical Crops Hainan University Haikou Hainan China; ^2^ Key Laboratory of Three Gorges Regional Plant Genetics & Germplasm Enhancement (CTGU)/Biotechnology Research Center China Three Gorges University Yichang Hubei China

**Keywords:** CIPK23, WHY transcription factor, abscisic acid, drought stress, cassava

Dear editor,

With the global climate change, drought has become one of the most serious environmental stresses that affect crop yield (Fahad *et al.*, 2017). As one of the most important food and energy crops, cassava (*Manihot esculenta*) feeds about 750 million people in the world, especially in Africa (Yan *et al.*, 2018). It is widely known that cassava is highly tolerant to drought and poor nutritional environment (De Souza *et al.*, 2017). However, the key regulators of drought response in cassava remain elusive. In our previous study, we have identified Whirly (MeWHY) transcriptional factors and revealed their roles in modulating plant disease resistance against cassava bacteria blight (CBB) through interacting with MeWRKY75 (Liu *et al.*, 2018). Herein, we further found that MeWHYs could physically interact with MeCIPK23 (Hu *et al.*, 2015; Yan *et al.*, 2018), as revealed by yeast two‐hybrid, biomolecular fluorescence complementation (BiFC), luciferase (LUC) complementation and pull‐down assays (Figure 1a‐d).

**Figure 1:**
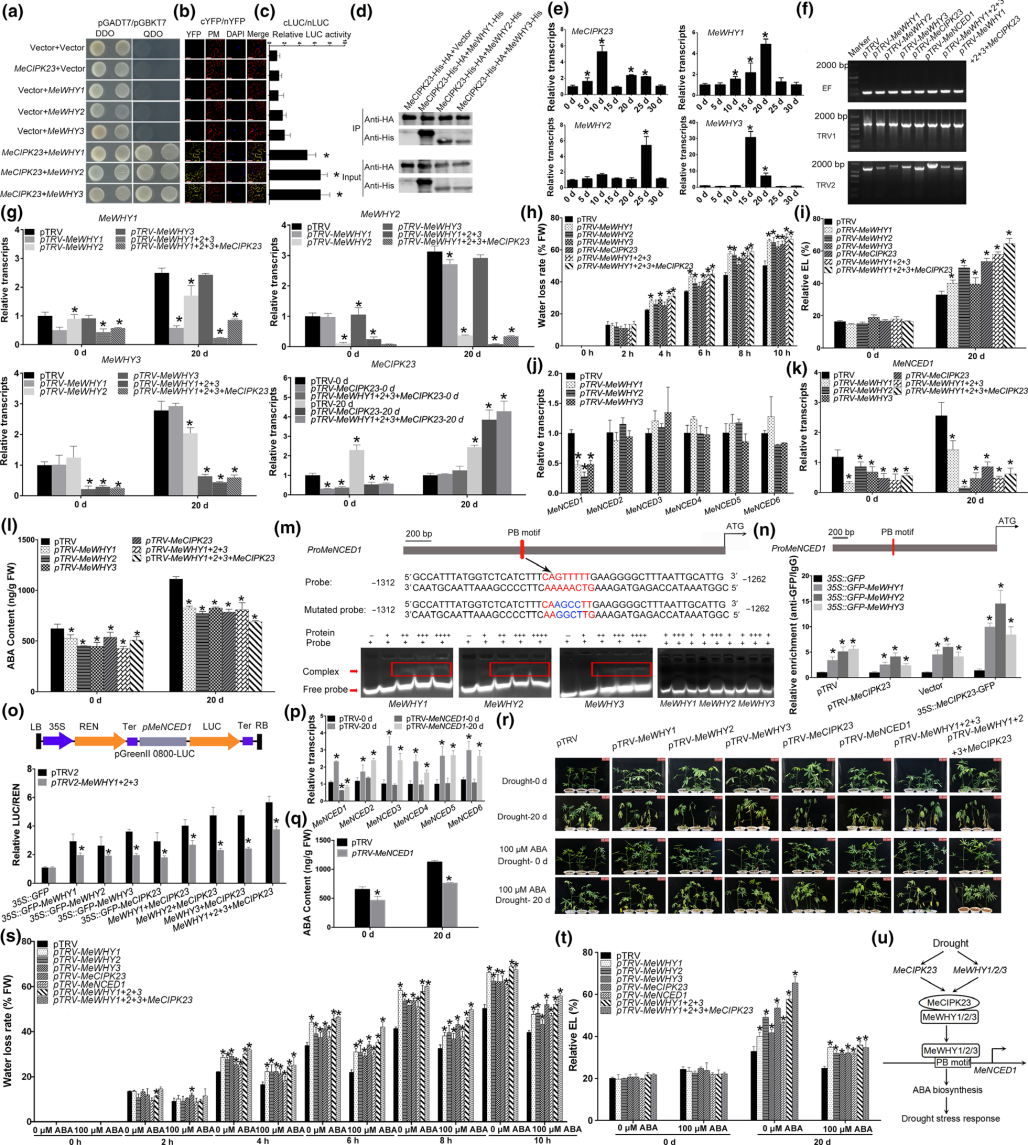
The interaction between MeCIPK23 and MeWHYs is essential for the activation of abscisic acid biosynthesis and drought stress resistance in cassava. (a)‐(d) Yeast two‐hybrid (a), BiFC (b), LUC complementation assay (c) and pull down (d) showing the physical interaction between MeCIPK23 and MeWHYs. DAPI‐stained cell nuclei and yellow fluorescent in BiFC were visualized using a confocal laser‐scanning microscope. Bar = 25 μm. (e) The transcript levels of *MeCIPK23* and *MeWHYs* in cassava leaves in response to drought stress. For the assay, plant leaves under control conditions (well‐watered) and drought stress conditions (with‐holding water) for indicated days were harvested. (f) RT‐PCR showing the expression of *TRV1* and *TRV2* in the VIGS plants. The reference gene *MeEF1a* and the viral transcripts TRV1/TRV2 were examined. (g) The transcript levels of corresponding genes in the gene‐silenced plant leaves. (h)‐(i) Water loss rate (h) and EL (i) in the gene‐silenced plant leaves in response to drought stress. (j) The transcript levels of *MeNCEDs* in the gene‐silenced plant leaves under control conditions. (k)‐(l) The transcript levels of *MeNCED1* (k) and the endogenous ABA accumulation (l) in the gene‐silenced plant leaves in response to drought stress. (m) EMSA showing the direct binding of MeWHYs to the probes of *MeNCED1* promoter. The sequences of control probe with PB motif and mutated probe with mutated PB motif are shown. The position of free probe and the protein‐probe complex are marked by arrow. (n) ChIP‐PCR showing the relative enrichment of MeWHYs in *MeNCED1* promoter. The same buffer without GFP antibody (IgG) was used as the native control of the GFP antibody. (o) Dual LUC assay showing the effects of MeWHYs and MeCIPK23 on the activity of *MeNCED1* promoter. (p) The transcript level of *MeNCED1* in the gene‐silenced plants. (q) The endogenous ABA accumulation in the gene‐silenced plants in response to drought stress. (r) Exogenous ABA restores the drought stress sensitivity of MeCIPK23‐MeWHYs‐MeNCED1 silencing plants. The pictures of different plants during drought stress conditions. Bars = 10 cm. (s)‐(t) Water loss rate (s) and EL (t) in the leaves in response to drought stress. (u) A proposed module of MeCIPK23‐MeWHYs‐MeNCED1 in drought stress response in cassava. In this study, cassava leaves were harvested for the assays. VIGS and gene overexpression in cassava leaves were performed through *Agrobacterium tumefacien*‐mediated transformation as we previously described (Liu et al., 2018; Zeng et al., 2019). All experiments were performed with at least three biological repeats. Statistical test was performed by SPSS. Kolmogorov–Smirnov test and Levene’s tests were used to check the normality of the data distribution and the variance homogeneity of the data, respectively. Asterisk symbols (*) suggested significant differences compared with control at *P *< 0.05.

WHY family proteins widely exist in plants and play multiple roles in modulating growth and development (Liu *et al.*, 2018; Prikryl *et al.*, 2008). In barley, WHY1 regulates drought stress‐induced leaf senescence through modulation of the expression of drought stress‐related genes and senescence‐related genes (Janack *et al.*, 2016). Previous study has identified a total of 25 *MeCIPKs* and shown that the transcripts of some *MeCIPKs* including *MeCIPK23* could be significantly regulated by drought stress and exogenous abscisic acid (ABA) treatment (Hu *et al.*, 2015). In addition, OsCIPK23 positively regulates plant drought stress resistance (Hu *et al.*, 2015). Based on previous studies (Hu *et al.*, 2015; Janack *et al.*, 2016) and the protein interaction between MeWHYs and MeCIPK23, their transcriptional levels in response to drought stress and their *in vivo* roles in plant drought stress resistance were investigated. The expression of *MeCIPK23* and *MeWHYs* were significantly and largely up‐regulated upon drought stress treatment at least at one time point (Figure 1e). The common induced transcripts of *MeWHYs* and *MeCIPK23* by drought stress in cassava indicated their possible involvement in plant drought stress response. Thereafter, we obtained MeWHYs‐ and MeCIPK23‐silenced cassava plants to silence single, triple or tetrad gene(s) via virus‐induced gene silencing (VIGS) (Zeng *et al.*, 2019; Figure 1f), and confirmed the decreased transcriptional levels of the corresponding genes but not other homologous genes in the silenced lines under both normal and drought stress conditions (Figure 1g). Compared with mock, gene‐silenced plants exhibited obvious drought stress sensitivity with more wilted leaves, higher water loss rate and higher electric leakage (EL) upon drought stress treatment for 20 day, and the effects are more obvious in triple‐ and tetrad‐silenced plants (Figure 1h, i, r). More wilted leaves, higher water loss rate and higher EL under drought stress conditions reflected worse leaf phenotype, lower water‐holding capacity and severer plasma membrane damage, respectively, suggesting that *MeWHY1*‐, *MeWHY2*‐, *MeWHY3*‐ and *MeCIPK23*‐silenced plants displayed enhanced drought stress sensitivity in cassava.

Abscisic acid plays a crucial role in plant drought stress resistance (Cai *et al.*, 2017). Therefore, we wondered whether *MeWHYs* and *MeCIPK23* regulated ABA level. We firstly detected the expression of *MeNCED* genes, which encode the key enzymes controlling ABA biosynthesis (Cai *et al.*, 2017). Because only the transcript of *MeNCED1* among six *MeNCEDs* exhibited a dramatic decrease in *MeWHYs*‐silenced cassava leaves (Figure 1j), this gene was selected for further analysis. Moreover, *MeWHYs‐* and *MeCIPK23*‐silenced cassava leaves had lower expression levels of *MeNCED1* after drought stress treated for 20 days in comparison to mock (Figure 1k). Consistent with compromised *MeNCED1* expression level, ABA content was also dramatically lower in *MeWHYs‐* and *MeCIPK23*‐silenced cassava leaves (Figure 1l).

Interestingly, we found a WHY‐binding PB motif existing in the promoter region of *MeNCED1* (Figure 1m), which has previously been suggested as the target of WHY proteins (Desveaux *et al.*, 2005; Liu *et al.*, 2018). Then, we analysed whether *MeNCED1* was a direct target of MeWHYs. Firstly, electrophoretic mobility shift assay (EMSA) indicated that MeWHYs could bind to the promoter region (−1312 to −1262) with PB motif of *MeNCED1*, since a second band with lower gel shift rate appeared and increased with the addition of MeWHY proteins (Figure 1m). However, MeWHYs could not bind to the mutated probe with mutated PB motif (Figure 1m), confirming that MeWHYs could specifically bind to the PB motif. In addition, ChIP‐PCR suggested that the promoter region of *MeNCED1* with PB motif was largely enriched by MeWHYs, and the enrichment levels were higher in *MeCIPK23* overexpressing background but lower in *MeCIPK23*‐VIGS background, indicating that MeCIPK23 could positively regulate the ability of MeWHYs to bind to PB motif (Figure 1n). Moreover, three MeWHYs could significantly activate the activity of *MeNCED1* promoter in dual LUC reporter system (Figure 1o). To sum up, these results suggested that *MeNCED1* is a direct target of MeWHYs. Notably, *MeCIPK23* overexpression could enhance the activity of *MeNCED1* promoter and enhance the effects of MeWHYs on activating the activity of *MeNCED1* promoter under mock conditions, but the effects of MeCIPK23 overexpression were significantly lower under *MeWHY1/2/3*‐VIGS background (Figure 1o), indicating that the interaction between MeCIPK23 and MeWHYs could direct regulate the activity of *MeNCED1* promoter. Consistently, we further constructed *MeNCED1‐*silenced plants by VIGS and found that the expression of *MeNCED1* but not other *MeNCEDs* (Figure 1p) and ABA content (Figure 1q) were attenuated in *MeNCED1‐*silenced cassava plants.

Exogenous application of 100 µm ABA enhanced plant drought stress resistance in wild‐type cassava plants (Figure 1r), and the drought stress sensitivity in *MeWHYs‐* and *MeCIPK23*‐silenced cassava plants could be restored by exogenous ABA treatment, at least partially (Figure 1r). Consistent with this, drought‐induced increase of water loss rate and relative EL was dramatically compromised by ABA treatment in *MeWHYs‐* and *MeCIPK23*‐silenced cassava leaves (Figure 1s‐t), indicating that ABA biosynthesis is directly involved in *MeWHYs‐* and *MeCIPK23‐* mediated drought stress resistance in cassava.

Taken together, we proposed a potential model for MeCIPK23‐MeWHYs‐mediated drought stress response in cassava (Figure 1u). Under drought stress conditions, the expression of *MeCIPK23* and *MeWHYs* are up‐regulated. In addition, MeCIPK23 interacts with MeWHYs, which directly bind to the PB element in the promoter of *MeNCED1* and activate its transcription. Then, the up‐regulated expression of *MeNCED1* results in elevated ABA biosynthesis and enhanced drought stress response. Therefore, this study provides new insight into the drought‐resistance mechanism in cassava and potential strategies for further crop breeding and germplasm enhancement.

## Conflict of interest

The authors declare no conflicts of interest.

## Authors’ contributions

Shi H conceived and directed this study, and revised the manuscript; Yan Y, Liu W and Wei Y performed the experiments, analysed the data, wrote and revised the manuscript.
